# Assessing the diagnostic utility of tRNA-derived fragments as biomarkers of head and neck cancer

**DOI:** 10.1016/j.tranon.2024.102135

**Published:** 2024-09-23

**Authors:** Matthew Uzelac, Weg M. Ongkeko

**Affiliations:** a Department of Otolaryngology-Head and Neck Surgery, University of California, San Diego, La Jolla, CA 92093, United States; b Research Service, VA San Diego Healthcare System, San Diego, CA 92161, United States; c Stanford University School of Medicine, Stanford, CA 94305, United States

**Keywords:** tRNA-derived fragment, Non-coding RNA, Head and neck cancer

## Abstract

Roughly 54,000 individuals are diagnosed with head and neck cancers in the United States yearly. Transfer RNA-derived fragments (tRF) are the products of enzymatic cleavage of precursor tRNAs, and have been proposed for use as biomarkers of head and neck cancer. In this study, we aim to further analyze the utility that tRFs might provide as biomarkers of head and neck cancer. tRF read counts were obtained for 453 tumor and 44 adjacent normal tissue samples and used to construct a gradient boosting diagnostic model. Although we identified 129 tRFs that were significantly dysregulated between these samples, the model achieved a sensitivity of only 69 % and a specificity of 59 %. tRFs are thought to induce the degradation of mRNA transcripts containing a complementary “seed” region. Despite the above performances, we chose to explore this concept of translational regulation by analyzing these tRFs for inverse correlation to the expression of select oncogenes and tumor suppressor genes implicated in head and neck cancer. Among others, CysGCA 5′-half and LysCTT 3′-tRF were upregulated in the tumor samples, and corresponded to decreased expression of PIK3R1, AKT1, and CPEB3. These transcripts were further found to contain numerous significantly complementary sites at which tRF-mediated mRNA degradation might occur. Although these tRFs did appear to correlate to many of the oncogenic metrics analyzed, we believe that additional research is needed before they might be used to improve the diagnosis, treatment, and survival of patients with this disease.

## Highlights

• 129 tRFs were found to be upregulated in head and neck tumor tissues.
• These tRFs correlated inversely to the expression of select tumor suppressor genes.
• A tRF-based diagnostic model achieved poor accuracies on an external cohort.

## Graphical abstract

**Figure:**
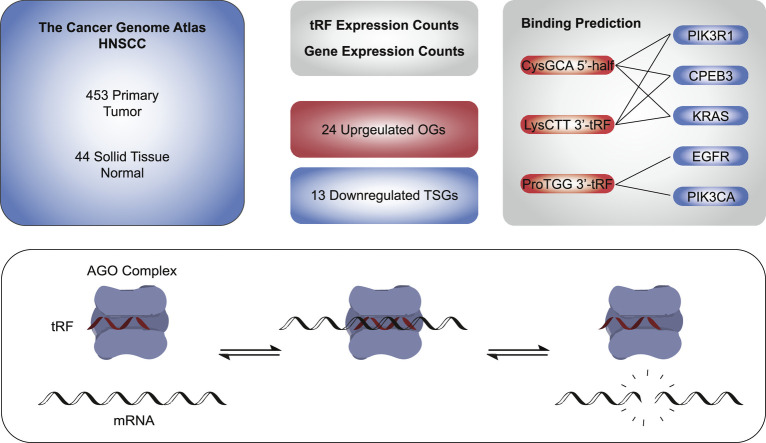

## Introduction

Transfer-RNA-derived fragments (tRFs) comprise a class of small non-coding RNAs. These molecules are derived from the enzymatic cleavage of precursor tRNAs, a process enriched in conditions of cellular stress [1,2]. Angiogenin, Dicer, and other ribonucleases are believed to perform this cleavage [[1], [2], [3], [4]]. Over recent years, tRFs have drawn increased attention for their transcriptional regulatory effects within cells [[5], [6], [7]]. tRFs are thought to act analogously to miRNAs by binding the Ago1, Ago3, and Ago4 proteins and traversing a cell to cleave endogenous mRNAs [[5], [6], [7]]. This mechanism of regulation is highly specific, and requires a precise “seed“ region of the tRF to contain near-perfect complementarity to the mRNA target of degradation [8]. Other mechanisms of regulation have been explored, including tRF-ribosome binding, though these are thought to have more diffuse effects on translation [[9], [10], [11]].

With the discovery of these effects, tRFs have seen increased investigation for their implications in human diseases, including cancers [[12], [13], [14], [15]]. In breast cancer, studies have shown specific tRFs to correspond to an increased degree of cellular invasion [16,17]. Select tRF have similarly been shown to promote a more aggressive phenotype and metastasis in colorectal cancers [18,19]. More recently, differential expression of tRFs has been used for cancer diagnosis. A study achieved 88.2 % accuracy in colorectal cancer diagnosis using GlyGCC 5′-tRF plasma levels [20]. A similar study achieved 79.9 % accuracy in breast cancer diagnosis using plasma tRFs [21]. Nonetheless, identifying the mechanistic relevance of tRFs to these cancers may further elucidate why these tRFs could be useful for diagnosis.

Head and neck cancers are believed to affect roughly 54,000 individuals in the United States yearly [22]. Risk factors for these cancers have been well-characterized, and largely include frequent alcohol use, tobacco use, and HPV infection [23,24]. However, early detection remains challenging. Genetic markers, both coding and non-coding, are the target of many diagnostic tests [25,26]. Of the non-coding markers, specific tRFs have been found to correlate to worsened prognosis and decreased survival in head and neck cancers [27,28]. The diagnostic utility of these tRFs has yet to be established. Thus, exploring the mechanistic relevance of tRFs in head and neck cancer may further support their use as biomarkers of this disease.

In this study, we analyzed the diagnostic utility of tRFs by comparing their expression between head and neck squamous cell carcinoma (HNSCC) tumor and adjacent normal tissue samples. tRF read counts were downloaded for 453 tumor and 44 normal tissue samples. We identified specific tRFs that were differentially expressed between these samples. We further identified inverse correlations between the expression of these tRFs and the expression of key oncogenes (OG) and tumor suppressor genes (TSG) in HNSCC. Binding analyses revealed sites at which these transcripts might bind with significant complementarity to induce Ago-mediated cleavage. These tRFs were used to construct a gradient boosting model designed to discern the tumor and adjacent normal tissue samples, which was validated on an external cohort. Ultimately, we hope this analysis provides additional insight into the role that tRFs might play as biomarkers of HNSCC. This may further our understanding of whether tRFs can provide a means for consistent and reliable diagnosis.

## Materials and methods

### Data acquisition

TCGA tRF read counts were downloaded from MINTbase v2.0 for 453 HNSCC tumor and 44 adjacent normal tissue samples (https://cm.jefferson.edu/mintbase-v2-0/, accessed on May 8, 2024). TCGA gene expression counts were downloaded from the GDC repository for these samples (https://portal.gdc.cancer.gov).

RNA-sequencing data of 14 oral squamous cell carcinoma and 7 normal tissue samples was downloaded from the NCBI's Sequencing Read Archive (https://www.ncbi.nlm.nih.gov/sra, accessed on Aug 25, 2024) under the accession PRJEB56345. tRF read counts were extracted from these samples using MINTmap v1.0 [29]. This software maps sequencing data to a reference database of tRF sequences intrinsic to the MINTmap software. These samples comprised an external validation cohort.

### Confounding etiology analyses

There are several major etiologies of HNSCC, each of which is known to exhibit a unique pathophysiology [23,24]. In order to assess the extent by which these etiologies might confound tRF expression, the microbiome R package was used to perform principal coordinal analyses (PCoA) for each of the following patient variables: alcohol consumption frequencies, smoking histories, HPV infection statuses, and anatomical tumor sites. This analysis reduced all of the tRF features in a sample into several arbitrary dimensions in order to visualize the extent by which tRF expression varies between patients of different alcohol consumption frequencies, for instance. Such tRFs should not be considered when analyzing tRF dysregulation between tumor and adjacent normal samples. PERMANOVA tests were used to determine whether each of these variables acted as a significant covariate toward tRF expression.

### Differential expression analyses

The Kruskal-Wallis test was used to identify tRFs that were differentially expressed between the cancer and normal samples (*p* < 0.05).

### Expressional correlation analyses

24 OGs and 13 TSGs that are implicated in HNSCC were selected from the COSMIC database and a literature review [30,31]. Spearmen's rank correlation coefficients were computed between the expression of each tRF and each gene. Only the tRFs that were significantly differentially expressed between the cancer and normal samples were analyzed.

### Binding affinity analyses

A collection of tRF-gene target binding interactions was downloaded from tRFTar [32]. This database houses an assembly of potential Ago-mediated tRF-gene target interactions that have been experimentally validated through crosslinking-immunoprecipitation and high-throughput sequencing datasets. Due to the degrading nature of tRFs, only the tRF-gene pairs of significantly inverse correlations were analyzed.

### tRF expression gradient boosting classifier

A gradient boosting model was created to discern the above tumor tissue samples from the adjacent normal tissue samples on the basis of their tRF expressions. This was achieved using the lightGBM R package. An initial model was constructed using the samples of the original TCGA cohort. Leave-one-out-cross validation (LOOCV) was used to assess the performance of this model, in which a single sample is excluded from the model's training. The model is then trained using the tRF expression values of the remaining samples and used to predict the disease state of the excluded sample. This process is repeated until all samples have been excluded once. LOOCV is less subject to intracohort variance than a traditional train-test split [33].

A model was then trained using the tRF expression values of all of the samples of the original cohort, then used to predict the disease states of the samples of the external validation cohort. For each of the above models, receiver operating characteristic (ROC) curves were created to display the models’ sensitivities at varying specificities. The area under the curve (AUC) for each of these plots was used as a metric of the models’ accuracies, with a value of 1.000 equating to perfect classification and a value of 0.500 equating to random classification.

### Plotting

Plots were generated using the R packages ggplot2, microbiome, pROC, and circlize.

## Results

### HNSCC confounding etiologies

**Fig. 1:**
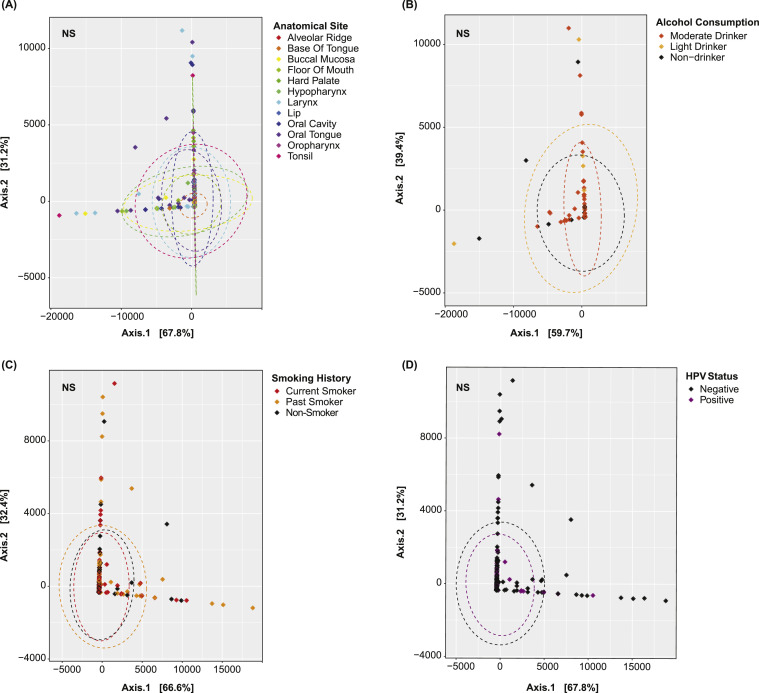
**Confounding Etiology Analysis** PCoA plots showing the tRF expressional profiles of patients with differing (A) anatomical tumor sites, (B) alcohol consumption frequencies, (C) smoking histories, and (D) HPV statuses. Points represent samples and colors represent their corresponding variable designations. Closer proximity indicates greater similarity in the samples’ tRF expressional profiles. Neither the anatomical tumor site or any of the above etiologies significantly related to tRF expression (NS – not significant).

There are several known etiologies of HNSCC, each of which is thought to exhibit a distinct pathophysiology from the others [23,24]. Differences in alcohol consumption frequencies, smoking histories, HPV infection statuses, and anatomical tumor sites may result in unique expressional patterns between samples [23,24]. Accordingly, PCoA models were constructed to assess the extent by which tRF expression varied with these etiologies and anatomical sites. These models attempt to simplify the read counts of all tRFs in a sample into several arbitrary dimensions. In these dimensions, a sample's proximity to another is indicative of wholistic similarities in their tRF expressional profiles. Samples extracted from different anatomical sites (Fig. 1A), patients with different alcohol consumption frequencies (Fig. 1B), patients with different smoking histories (Fig. 1C), and patients with and without HPV (Fig. 1D) appear to be of overlapping proximity, suggesting that these variables do not confound tRF expression. Indeed, tRF expression was found to not vary significantly with these covariates (PERMANOVA, *p* < 0.05).

### Differentially expressed tRFs

**Fig. 2:**
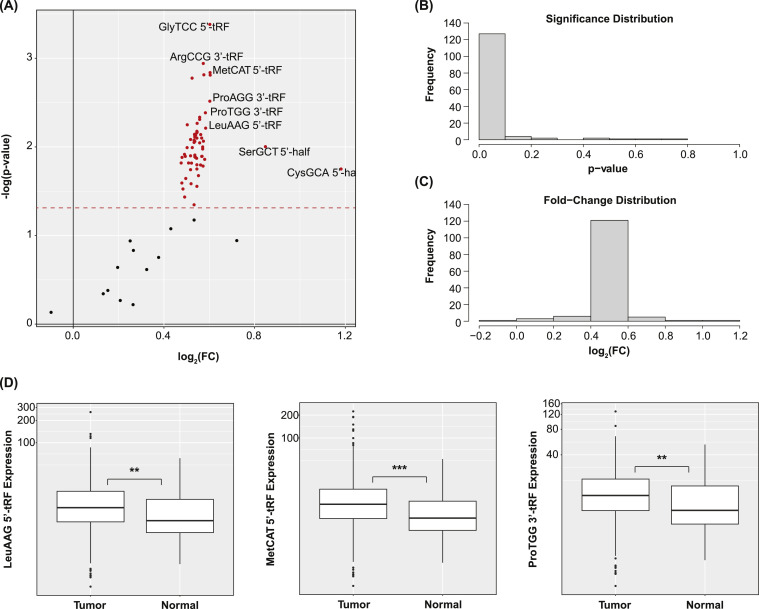
**Differentially Expressed tRFs** (A) Volcano plot showing differentially expressed tRFs. Points represent tRFs. Fold-change values between cancer and normal samples are shown alongside their corresponding test statistics. The red line signifies *p* < 0.05. 129 tRFs were determined to be significantly dysregulated between these samples. (B) Histogram of test statistics. (C) Histogram of fold-change values. (D) Box plots showing the expression of LeuAAG 5′-tRF, MetCAT 5′-tRF, and ProTGG 3′-tRF between the tumor and adjacent normal samples (** *p* < 0.01, *** *p* < 0.001).

The Kruskal-Wallis test was used to identify tRFs that were differentially expressed between the tumor and adjacent normal samples. 129 tRFs were identified (Fig. 2A). CysGCA 5′-half, LeuAAG 5′-tRF, and ProTGG 3′-tRF were considerably upregulated. A sizable portion of all of the tRFs investigated was significantly dysregulated (Fig. 2B). All but one were upregulated in the tumor samples (Fig. 2C). This is consistent with the notion that tRFs are formed in conditions of cellular stress [1,2]. Select box plots were created to visualize the expression of LeuAAG 5′-tRF, MetCAT 5′-tRF, and ProTGG 3′-tRF between the tumor and adjacent normal tissue samples (Fig. 2D). Only the tRFs that were significantly differentially expressed between the tumor and adjacent normal samples were considered for further analysis.

### tRF expressional correlations to HNSCC oncogenes and tumor suppressor genes

**Fig. 3:**
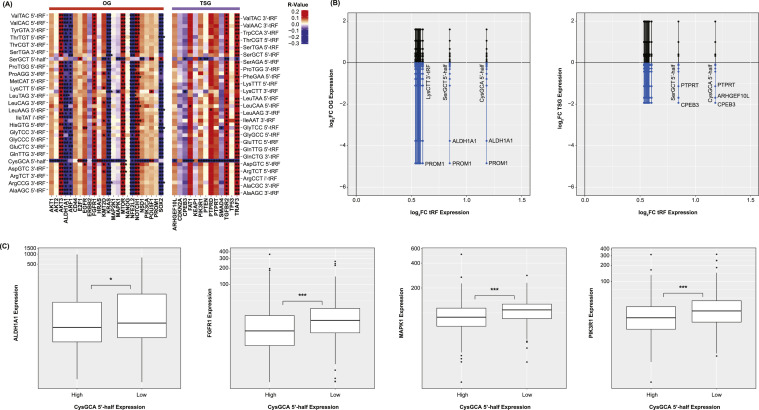
tRF Expressional Correlations to HNSCC Oncogenes and Tumor Suppressor Genes (A) Heatmap of tRF-OG and tRF-TSG correlations. Fill colors are correlation coefficients (R-values). CysGCA 5′-half and SerGCT 5′-half were found to inversely correlate to the expression many of the OGs and TSGs studied. (B) Lollipop plots showing the fold-changes in OG (left) and TSG (right) expression with respect to the fold-change in each tRF's expression. A tRF with a positive fold-change and a negative correlation to a TSG indicates downregulation of that TSG in cancer samples (blue). (C) Box plots showing the expression of ALDH1A1, FGFR1, MAPK1, PIK3R1 between samples with “high” and “low” expression of CysGCA 5′-half. Samples were binarized based on their expression of CysGCA 5′-half in relation to the median expression of CysGCA 5′-half across all samples (* *p* < 0.05, ** *p* < 0.01, *** *p* < 0.001).

The differentially expressed tRFs were analyzed for correlation to 24 OGs and 13 TSGs known to be implicated in HNSCC (see Materials and Methods). The tRF-Ago complex is known to degrade mRNA. As such, a particular emphasis was placed in anticorrelated tRF-gene pairs. Spearman's correlations were computed for each tRF-gene pair (Fig. 3A). CysGCA 5′-half and SerGCT 5′-half appeared to correlate significantly to many of the genes studied. These tRFs were found to be upregulated in the cancer samples, correlating to decreased expression of several TSGs, including TP53, PIK3R1, and CPEB3. Several tRFs that were upregulated in tumor samples were also found to correlate inversely to the expression of the OGs studied, including PIK3CA and KRAS. These results are consistent with the notion of Ago-mediated degradation, though they are purely correlational. The fold-change in expression of each OG and TSG was further calculated between the tumor and adjacent normal samples. These fold-changes were plotted against the fold-changes in each tRF's expression (Fig. 3B). Among other observations, an increase in CysGCA 5′-half and SerGCT 5′-half expression corresponded to a decrease in CPEB3 and PTPRT expression. For each of the significant correlations above, the samples were binarized based on their expression of the component tRF in relation to the median expression of that tRF across all samples. Box plots were created to visualize the expression of ALDH1A1, FGFR1, MAPK1, PIK3R1 between samples with “high” and “low” expression of CysGCA 5′-half (Fig. 3C). Samples with greater expression of CysGCA 5′-half contained lesser expression of these genes, suggesting the two are inversely correlated.

### tRF-gene pairs are sufficiently complementary for degradation

**Fig. 4:**
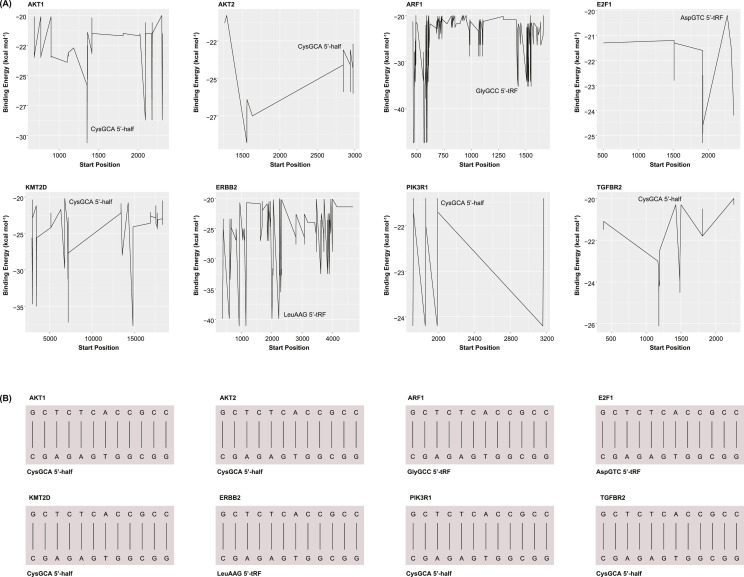
Binding Affinities Between Significant tRF-Gene Pairs (A) Select line plots showing the start position at which each tRF-gene heteroduplex may assemble. Positions describe the starting base pair along the OG or TSG, and not the tRF. The binding energy at each site is shown. (B) Complementary base pairs that comprise each tRF-gene heteroduplex. Straight connections indicate Watson-Crick base pairs. Dotted connections indicate non-canonical base pairs.

Complementarity of a 7 to 9 nucleotide “seed” region is necessary for the Ago complex to degrade a mRNA transcript [8]. Statistical binding affinities were obtained for each tRF-gene target pair from tRFTar (see Materials and Methods) [32]. Only the pairs of significantly inverse correlations were analyzed. Select tRF-gene pairs were found to contain multiple sites of sufficient complementarity for Ago-mediated cleavage, including CysGCA 5′-half-PIK3R1, CysGCA 5′-half-ARF1, and LeuCAA 5′-tRF-CPEB3. The precise binding sites were visualized for select genes (Fig. 4A). These plots show the base pair positions along each gene at which the start of the tRF heteroduplex may form. The energy of heteroduplex formation is shown for each site. Notably, CysGCA 5′-half was found to contain numerous binding sites along many of these genes, including PIK3R1 and AFR1. For each tRF-gene pair, the site of the most negative binding energy was selected. The complementary base pairs that comprise each of these heteroduplexes were visualized (Fig. 4B). After being loaded with the appropriate tRF, the Ago complex may bind to these sites with sufficient complementarity to cleave the OG or TSG transcript,

### Creation of a tRF-based diagnostic classifier

**Fig. 5:**
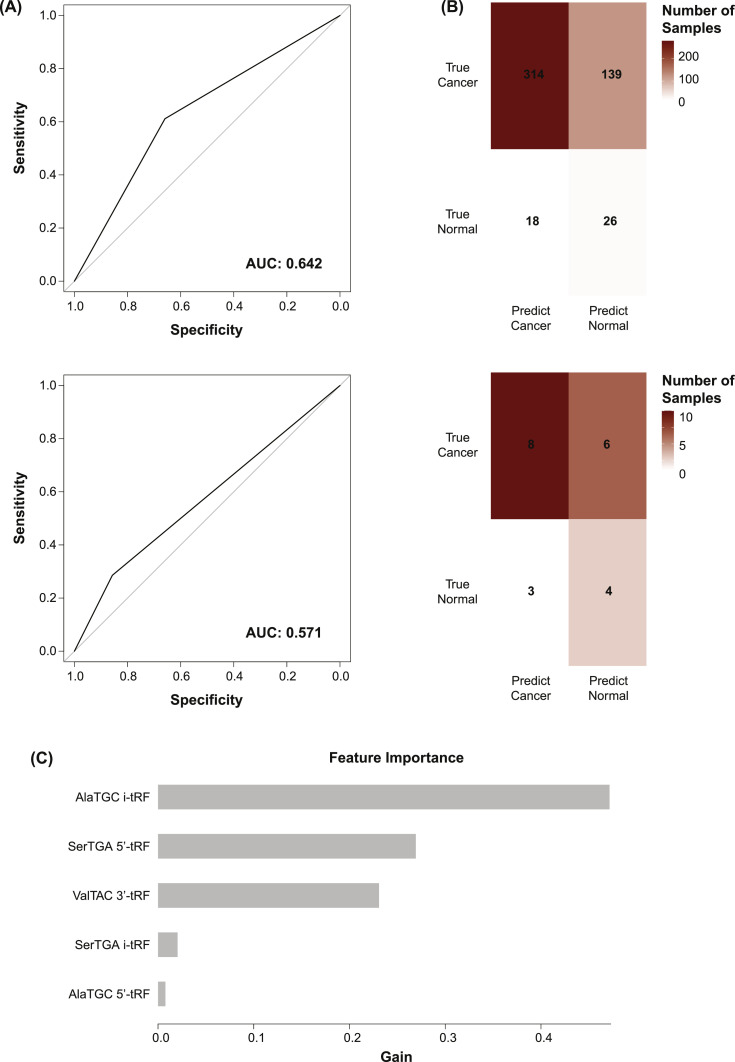
tRF Expression-Based Classifier (A) ROC curves showing the classifier's performance for the primary (top) and external validation (bottom) cohorts. The classifier was constructed using the expression values of 161 tRFs. Plots show the model's sensitivity at varying specificities. AUCs are a metric of the model's performance, with an AUC of 1.000 equating to the correct prediction of all patients’ responses and an AUC of 0.500 equating to random guesses. (B) Confusion matrices showing the true distribution of samples and the distribution of the model's predictions for the primary (top) and external validation (bottom) cohorts. The model incorrectly classified the disease states of a considerable portion of samples. (C) Bar chart showing the gain provided to the classifier from each of its top five most important features. Model gain is a metric of feature importance.

Despite the genetic interactions suggested of the above tRFs through tRFTar [32], it is unclear whether these tRFs might provide clinical benefit as biomarkers. We constructed a gradient boosting model designed to discern the above HNSCC tumor tissue samples from the adjacent normal tissue samples on the basis of their tRF expressions. Although the use of tissue samples cannot provide the same degree of clinical utility as blood or saliva, we hope that such a model will reveal the practicality of downstream blood- or saliva-based diagnostic models. 161 tRFs were identified across the 453 tumor tissue and 44 adjacent normal tissue samples of the original TCGA cohort. The expression values of these tRFs were used to construct an initial model. Using LOOCV (see Materials and Methods), the model achieved an accuracy of 68 % and an AUC of 0.642 (Fig. 5A). This corresponded to a sensitivity of 69 % and a specificity of 59 % (Fig. 5B). When the model was applied to an external validation cohort of 14 oral tumor and 7 normal tissue samples, an accuracy of 57 % and an ROC of 0.571 were observed (Fig. 5A). This corresponded to a sensitivity of 57 % and a specificity of 57 % (Fig. 5B). AlaTGC i-tRF, SerTGA 5′-tRF, and ValTAC 3′-tRF were among the most important features to the model (Fig. 5C). Nonetheless, this specific panel of tRFs appeared to provide only slight diagnostic utility for the HNSCC tumor tissues analyzed.

**Fig. 6:**
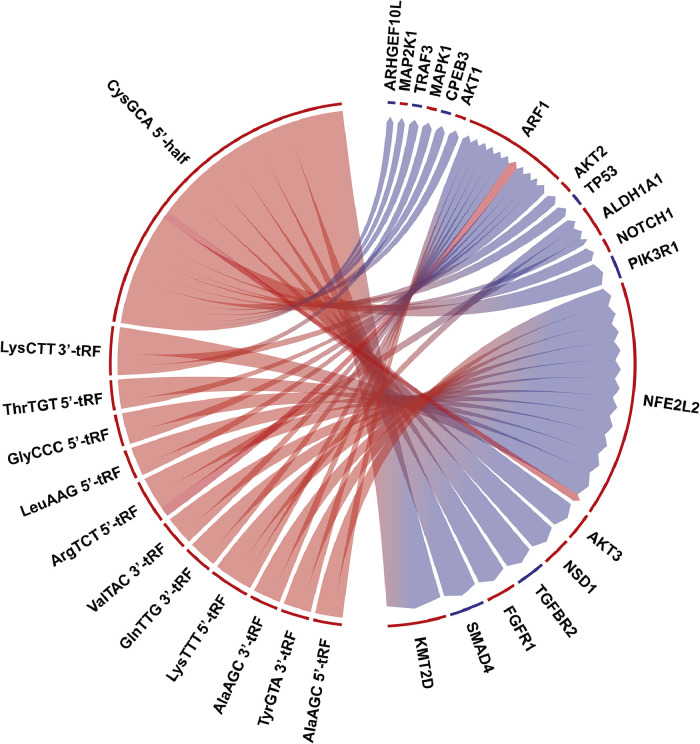
**tRF-Mediated Transcriptional Regulations** Chord diagram of significant tRF-gene pairs. Connection widths were calculated as the products of the negative logarithms of each of the significance in each tRF's differential expression between the tumor and normal samples and the significance in each tRF's correlation to the OGs and TSGs studied. The bordering colors indicate whether each tRF or gene was upregulated (red) or downregulated (blue) in the tumor tissue samples.

As a composite of the above analyses, tRF-gene pairs were compiled based on the significance in each tRF's differential expression between the tumor and normal samples and the significance in each tRF's correlation to the OGs and TSGs studied. A chord diagram was created to visualize these pairs (Fig. 6). The width of each connection reflects the product of the negative logarithms of the above significance metrics. CysGCA 5′-half and LysCTT 3′-tRF, both upregulated in cancer samples, showed prominent connections to the OG NFE2L2. These tRFs were also strongly connected to the TSGs SMAD4 and PIK3R1. The pairs of this diagram thoroughly summarize our findings in this study, although these tRFs appeared only slightly capable of discerning HNSCC tumor and normal tissues. Given the genetic interactions above, it is clear that additional research is required to determine the pathophysiological role that these tRFs might play in HNSCC.

## Discussion

Given the results above, we believe that tRFs cannot provide the level of diagnostic utility for HNSCC as that achieved by similar studies of other cancer types. Studies have used tRF expression levels to discern the plasma samples of cancer patients from those of healthy controls with reasonable accuracies [20,21]. However, these studies fail to validate their classifiers using external cohorts. Indeed, when our classifier was applied to an external cohort, it's accuracy fell from 68 % to 57 %. The panel of tRFs used to discern the original tumor and adjacent normal tissue samples was not generalizable to the external tumor and normal tissue samples. Such generalizability is necessary of a biomarker.

Despite our classifier's performance, we identified 129 tRFs that were significantly differentially expressed between the tumor and normal tissues of the original dataset. Several of these tRFs have been previously shown to correlate to worse measures of prognosis and survival in HNSCC, including LysCTT 3′-tRF [27,28]. However, we have noted several inconsistencies with our study compared to others, such as the importance of CysGCA 5′-half [27,28]. In addition to the lack of generalizability describe above, we believe that differences in the chosen tRF stratification methods may have influenced this; we chose to group tRFs based solely on their anticodon sequence, while other studies opted to use MINTbase's unique tRF identifiers. These identifiers contain information on the precise position at which each tRNA was fragmented. Unless the flanking base pairs are directly required to bind a specific mRNA transcript, we believe that these differences are trivial. Moreover, sequencing and mapping such small fragments is unlikely to provide precision at the level of a few flanking base pairs. For these reasons, we believe that additional research is necessary before tRFs might be implemented as biomarkers for diagnosis.

We found that many of these tRFs correlated inversely to the expression of key OGs and TSGs implicated in HNSCC. Our discovery of precise binding sites between these transcripts might suggest the likelihood of Ago-mediated degradation, potentially contributing to the process of oncogenesis. We should note that these tRFs are not inducing a complete and extensive knockout of the above OGs and TSGs. Rather, we propose that these expressional correlations are slight, and may only moderately alter oncogenic regulatory pathways within a cell. This is in accordance with the poor performance observed of our classifier. One might expect a strong pathophysiological driver to serve as a robust biomarker. It should also be noted that these observations are purely correlational, and that additional *in vitro* research may confirm the role that these tRFs play in HNSCC.

CysGCA 5′-half and LysCTT 3′-half were found to correlate to decreased expression of many of the OGs and TSGs studied. These tRFs were more highly expression in the tumor tissues. Given the inverse correlations and binding sites identified above, these tRFs may plausibly discourage translation of these TSGs. PIK3R1, for instance, is known to have decreased expression in the tumors of HNSCC patients and has been shown to suppress tumor growth [34,35]. Greater expression of CysGCA 5′-half was found to correlate to decreased expression of this gene, and numerous binding sites were identified between these transcripts at which the Ago complex might assemble. Theoretically, CysGCA 5′-half-mediated degradation may hinder the regulatory effects of PIK3R1 that might otherwise be observed [34,35]. Further experimentation should be used to confirm this notion *in vitro*.

It should also be noted that several tRFs correlated inversely to the expression numerous OGs. Several of these tRF-OG pairs were found to contain sufficiently complementary sites at which the Ago complex might assemble, including CysGCA-PIK3CA, CysGCA-AKT1, and CysGCA-KMT2D. These tRFs were upregulated in the tumor samples, with greater expression correlating to decreased expression of these OGs. Theoretically, Ago-mediated degradation of these transcripts might exert a protective effect in tumors. This effect would be rather slight, however, as these OGs were upregulated in cancer samples as a whole. Again, further experimentation *in vitro* might reveal the relevance of these pairs to HNSCC.

We hope that this study might also provide insight beyond the analyses themselves. Our findings might suggest that the transcriptome is highly fluid and variable among different tumor microenvironments. The inconsistencies in our results might speak to the complexity of the non-coding landscape of a cell. tRFs did appear to correlate to many of the oncogenic metrics analyzed, albeit less consistently than many of the coding elements known to literature. Moreover, tRFs only compose a fraction of the non-coding RNAs known to exist. This study may further suggest the importance of non-coding RNAs as a whole in oncogenesis. Indeed, other studies have attempted characterize the effects of the non-coding landscape in HNSCC [26,36]. The non-coding landscape is a complexity of the tumor microenvironment whose unraveling might serve to advance precision medicine.

Ultimately, we hope that this study reveals the complexity and caution that should be taken in using tRFs for diagnosis. Extensive validation must be used to determine whether a tRF might be generalized as a biomarker for a given cancer type. Although we identified numerous expressional correlations between the tRFs, OGs, and TSGs studied, these tRFs provided only slight diagnostic utility for HNSCC. Further investigation might be used to create an improved diagnostic panel, though it is unclear how the results above might translate to a plasma- or saliva-based model. We do hope that this study further speaks to the relevance of the non-coding landscape to oncogenesis. Perhaps by further demystifying the non-coding interactions of a cell, we may learn which aspects might be harnessed for improved diagnosis, treatment, and survival of patients with HNSCC.

We must note that our results are limited, largely due to the correlational nature of the above analyses. Though we do not demonstrate a causal relationship between tRFs and HNSCC pathogenesis, we hope that this study speaks to the relevance of tRFs to this disease. We'd also like to note that the analyzed samples span all pathologic stages. The creation of a diagnostic panel using only early-stage cancers would prove highly useful for early diagnosis. However, given the current performance of classifier, it is likely that an early-stage classifier would be even less capable at capturing relevant differences in tRF expression between cancerous and non-cancerous tissue.

## Data availability statement

All TCGA tRF read counts can be downloaded from MINTbase v2.0 (https://cm.jefferson.edu/mintbase-v2-0/). All TCGA gene expression counts can be downloaded from the GDC repository (https://portal.gdc.cancer.gov/repository).

All RNA-sequencing data can be downloaded from the NCBI's Sequencing Read Archive (https://www.ncbi.nlm.nih.gov/sra) under the accession PRJEB56345.

## Funding

This work was supported by the UC San Diego Academic Senate Grant RG096651 to W.M.O.

## CRediT authorship contribution statement

**Matthew Uzelac:** Writing – review & editing, Writing – original draft, Visualization, Software, Methodology, Investigation, Formal analysis, Data curation. **Weg M. Ongkeko:** Writing – review & editing, Supervision, Resources, Project administration, Investigation, Funding acquisition, Data curation, Conceptualization.

## Declaration of competing interest

The authors declare that they have no known competing financial interests or personal relationships that could have appeared to influence the work reported in this paper.
